# Animal Models of PTSD: The Socially Isolated Mouse and the Biomarker Role of Allopregnanolone

**DOI:** 10.3389/fnbeh.2019.00114

**Published:** 2019-06-11

**Authors:** Graziano Pinna

**Affiliations:** The Psychiatric Institute, Department of Psychiatry, College of Medicine, University of Illinois at Chicago, Chicago, IL, United States

**Keywords:** post-traumatic stress disorder, translational neuroscience, social isolation, biomarker axis, neurosteroids, endocannabinoids, PTSD rodent models, PTSD treatments

## Abstract

Post-traumatic stress disorder (PTSD) is a debilitating undertreated condition that affects 8%–13% of the general population and 20%–30% of military personnel. Currently, there are no specific medications that reduce PTSD symptoms or biomarkers that facilitate diagnosis, inform treatment selection or allow monitoring drug efficacy. PTSD animal models rely on stress-induced behavioral deficits that only partially reproduce PTSD neurobiology. PTSD heterogeneity, including comorbidity and symptoms overlap with other mental disorders, makes this attempt even more complicated. Allopregnanolone, a neurosteroid that positively, potently and allosterically modulates GABA_A_ receptors and, by this mechanism, regulates emotional behaviors, is mainly synthesized in brain corticolimbic glutamatergic neurons. In PTSD patients, allopregnanolone down-regulation correlates with increased PTSD re-experiencing and comorbid depressive symptoms, CAPS-IV scores and Simms dysphoria cluster scores. In PTSD rodent models, including the socially isolated mouse, decrease in corticolimbic allopregnanolone biosynthesis is associated with enhanced contextual fear memory and impaired fear extinction. Allopregnanolone, its analogs or agents that stimulate its synthesis offer treatment approaches for facilitating fear extinction and, in general, for neuropsychopathologies characterized by a neurosteroid biosynthesis downregulation. The socially isolated mouse model reproduces several other deficits previously observed in PTSD patients, including altered GABA_A_ receptor subunit subtypes and lack of benzodiazepines pharmacological efficacy. Transdiagnostic behavioral features, including expression of anxiety-like behavior, increased aggression, a behavioral component to reproduce behavioral traits of suicidal behavior in humans, as well as alcohol consumption are heightened in socially isolated rodents. Potentials for assessing novel biomarkers to predict, diagnose, and treat PTSD more efficiently are discussed in view of developing a precision medicine for improved PTSD pharmacological treatments.

## Introduction

Post-traumatic stress disorder (PTSD) is a multifaceted psychiatric disorder characterized by a high worldwide prevalence in the general population and a consistent global burden and disability. In the U.S., about 50%–85% of individuals during their lifetime experience traumatic events, of these, about 6.8% develop PTSD (Kessler et al., 2005). However, its prevalence is even higher, reaching 25%–50%, in individuals exposed to warzones or in victims of domestic violence and abuse, including children and battered women, respectively (Goldstein et al., 2016). Importantly, women are particularly susceptible to develop PTSD as compared with men (Shansky, 2015; Yehuda et al., 2015). Other predictors for developing PTSD, include characteristics of the traumatic event for a given exposed individual (Bichescu et al., 2005). Comorbidity with other psychiatric disorders, such as major depressive disorder, anxiety spectrum disorders, and alcohol use disorder (AUD), or with suicide, as well as, overlapping of symptoms with these disorders are very common in individuals affected by PTSD (Shalev, 2001; Lassemo et al., 2017; Gagne et al., 2018). Together, these complications result in a general difficulty in diagnosing PTSD and make treatment selection difficult (Greene et al., 2016). Current pharmaco-treatment for PTSD relies in the administration of the selective serotonin reuptake inhibitors (SSRIs), such as paroxetine and sertraline, the only FDA-approved drugs for PTSD (Friedman and Bernardy, 2017). These drugs are associated with poor response rate in a consistent number of treatment-seeking patients, with active military members and veterans who are relatively non-responsive to SSRIs (Bernardy and Friedman, 2015; Starke and Stein, 2017). Developing suitable animal models for PTSD and discovering reliable biomarkers that allow a more accurate diagnosis, based on objective measures, may improve quality of healthcare. Biomarker discovery will indeed permit developing targeted drugs and may generally offer more treatment options, which is highly desirable and needed (discussed in Aspesi and Pinna, 2018).

While a number of animal models in mice and rats were developed in the past decades that, at least, partially recapitulate several neurochemical and behavioral deficits encountered in the wide ranging PTSD symptoms clusters, none of them is currently recognized as an optimal match with the human neuropathology (reviewed in Aspesi and Pinna, 2019). However, some of them reproduce core aspects of PTSD, including deficits in fear extinction and fear extinction retention and even transdiagnostic aspects relevant for comorbidity with depression, suicide and AUD. Notwithstanding sex matters with PTSD, sex as a biological variable in research including females has only recently being intensified and the sex-effect or the effect of the menstrual cycle or pregnancy in women with PTSD only recently has been taken into examination (Onoye et al., 2013; Pineles et al., 2017, 2018). In rodent PTSD models, these sex-related effects were scantily studied with very few studies that have attempted to reproduce endophenotipic expression of female PTSD neurobiology into female rodents (Cohen and Yehuda, 2011; reviewed in Keller et al., 2015; Aspesi and Pinna, 2019). Hence, sex-related studies in PTSD neurobiology are urgent and a priority in both clinical and preclinical research.

Furthermore, to add to the general complexity and heterogeneity of PTSD, it is conceivable that factors, including the type and the duration in time of a traumatic event, as well as, the condition of individuals in a given time when they are exposed to trauma, altogether, may dictate the development of subtypes of PTSD (Stein et al., 2016). Collectively, all these factors are important aspects that may drive establishing successful PTSD animal models. Often, the question arises as to whether an experimental model of PTSD should exclusively recapitulate core traits of PTSD, such as extinction deficits and avoidance or rather should take into account what is often encountered in the diagnosis of PTSD patients, for example, comorbidities with other mental disorders (discussed in Aspesi and Pinna, 2019).

The recent progress that has been made in understanding PTSD neurobiology has facilitated the development of experimental stress-induced animal models (Torok et al., 2018). However, PTSD remains a neuropathology with no specific pharmacological treatments, no established and reliable biomarkers, and PTSD animal models only reproduce PTSD neurobiology to a limited degree. While previous recent articles examined a number of animal models of PTSD and the validity of several biomarker candidates that have been proposed for PTSD (Aspesi and Pinna, 2018, 2019), this review will focus on the socially isolated mouse model of stress-induced fear extinction deficits. Other abnormal behavioral deficits will be discussed as well as commonalities with PTSD neurobiology in humans, such as reproducing endophenotipic features observed in PTSD patients. Transdiagnostic aspects shared with depression, anxiety, suicide and AUD are also discussed. This review article also analyses running findings suggesting the neurosteroid, allopregnanolone biosynthesis and its targets may prove valuable for establishing a *biomarker axis* suitable for PTSD. It is conceivable that allopregnanolone may play a key role to predict, diagnose and suggest an optimal treatment selection for PTSD in the near future.

## Allopregnanolone From Its Discovery in Adrenal Glands to a Role in Mood Disorders

**Figure 1:**
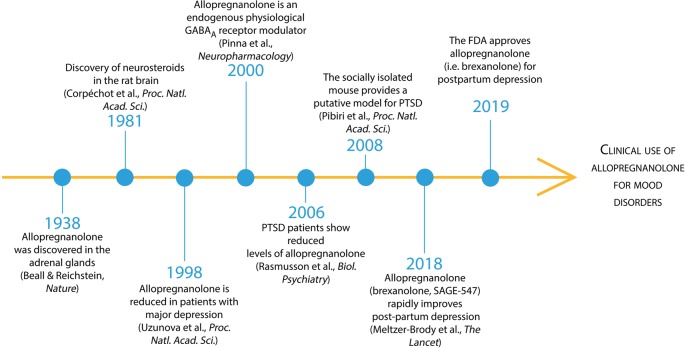
Timeline of allopregnanolone from its discovery to FDA preapproval for the treatment of mood disorders. Beall and Reichstein discovered allopregnanolone in 1938 in the adrenal glandswhere 5α-reductase metabolizes progesterone into 5α-dihydroprogesterone and then the enzyme 3α-hydroxysteroid dehydrogenase produces allopregnanolone (Beall and Reichstein, 1938). In 1981, Baulieu’s team discovered that the brain “acting like a peripheral gland” synthetize allopregnanolone *de novo* starting from pregnenolone, the precursor of all neurosteroids (Corpéchot et al., 1981). Allopregnanolone’s pharmacological effects following its administration in animal models and humans are mediated by the fast allosteric modulation of the action of GABA at GABA_A_ receptors (Majewska et al., 1986; reviewed in Belelli et al., 2009). The neurophysiological role of allopregnanolone in fine-tuning GABA_A_ receptors to agonists, positive allosteric modulators, and GABAmimetic agents, was unveiled thereafter (Pinna et al., 2000). Allopregnanolone levels were found decreased in mood disorders, including major unipolar depression and PTSD (Romeo et al., 1998; Uzunova et al., 1998; Rasmusson et al., 2006, 2019).An animal model of stress-induced behavioral dysfunction, including fear extinction deficits and aggressive behavior associated with a corticolimbic allopregnanolone biosynthesis downregulation was proposed therein after (Pinna et al., 2008; Pibiri et al., 2008). More recently, phase 3 clinical trials have established the clinical relevance of allopregnanolone in mood disorders (Kanes S. J. et al., 2017; Meltzer-Brody et al., 2018). Intravenous allopregnanolone (brexanolone or SAGE-547) or an orally-active, allopregnanolone’s analog (SAGE-217), showed a rapid and long-lasting remission of post-partum depression and major depressive disorder symptoms, respectively. These successful clinical trials led to the FDA approval of brexanolone for the treatment of post-partum depression in March 2019 and encouraged the possible future clinical use of brexanolone or SAGE-217 for the treatment of mood disorders, including PTSD.

Following its discovery in 1938 by Beall and Reichstein in the adrenal glands (Figure 1), allopregnanolone was recognized as a 5α-reduced metabolite of progesterone (Beall and Reichstein, 1938). It was named a *neurosteroid* in 1981 by Baulieu’s team who discovered that the brain “acting like a peripheral gland,” expresses the enzymatic machinery required to synthetize allopregnanolone *de novo* starting from pregnenolone, the precursor of all neurosteroids (Corpéchot et al., 1981). Allopregnanolone’s anti-convulsant, anxiolytic and anti-depressant pharmacological effects after its administration in animal models and humans were soon recognized to be mediated by a mechanism of action that includes the fast allosteric modulation of the action of GABA at GABA_A_ receptors (Majewska et al., 1986; reviewed in Belelli and Lambert, 2005; Belelli et al., 2009, 2018). In the year 2000, the neurophysiological role of allopregnanolone in permitting the fine-tuning and regulating the strength of GABA_A_ receptors to agonists, positive allosteric modulators, and GABAmimetic agents, was unveiled (Pinna et al., 2000). By acting at GABA_A_ receptors, allopregnanolone also regulates emotional behavior in rodent stress models of behavioral abnormalities and humans with PTSD and major unipolar depression (Uzunova et al., 1998; Pinna et al., 2003, 2008; Rasmusson et al., 2006, 2019; Pineles et al., 2018). More recently, several phase 3 clinical trials have established the clinical relevance of allopregnanolone in mood disorders. Intravenous allopregnanolone (brexanolone or SAGE-547) or an orally-active, allopregnanolone’s analog, named SAGE-217, showed a rapid and long-lasting remission of post-partum depression and major depressive disorder symptoms, respectively (Kanes S. J. et al., 2017; Kanes S. et al., 2017; Meltzer-Brody et al., 2018,^1^). These studies, in March 2019, led to the FDA approval of allopregnanolone (i.e., brexanolone) as the first specific treatment for post-partum depression that will allow this “endogenous tranquillizer” to be prescribed as a novel treatment for mood disorders starting in Summer 2019. On the other hand, if successfully developed, SAGE-217 will be the first durable, rapid-acting, oral, short-course treatment for mood disorders and potentially may be applied to test whether administered during prolonged exposure therapy for PTSD, it facilitates recovery in patients. The new generation of *rapid-acting antidepressants* has just emerged and may likely dominate the field of neuropsychopharmacology for the next decades to come.

The finding that the traditional gold-standard treatment option for PTSD, the selective serotonin reuptake inhibitors (SSRIs), is efficient in about half of the treated patients (reviewed in Golden et al., 2002; Rush et al., 2006; Kemp et al., 2008; Bernardy and Friedman, 2015), suggests that mood disorders emerge from complex neurobiological backgrounds and only one molecular deficit may not reflect a valid biomarker for the disorder under examination. Likewise, only one treatment cannot be the answer to improve symptoms in all patients, following the one-fit-all treatment expectation (Brewin, 2001; Aspesi and Pinna, 2018). Overall, discovering biomarkers that may lead to precision medicine for PTSD is in high demand. Novel advances in the field have been possible by employing state-of-the-art technologies and more reliable animal models (reviewed in Ngounou Wetie et al., 2013; Aspesi and Pinna, 2018, 2019). However, more research is needed to establish a reliable biosignature for PTSD and other mood disorders.

## Allopregnanolone: A Biomarker Candidate and a Treatment Endpoint for Mood Disorders

Research for effective biomarkers in psychiatric disorders still remains backward when compared to most fields of medicine that heavily rely on biomarkers for their prediction, prevention, diagnosis and assessment of the most effective treatments (discussed in Fernandes et al., 2017; Aspesi and Pinna, 2018). Diagnosis of PTSD and mood disorders still rely on subjective measures, including questionnaires and description of symptoms by the patients to the psychiatrist or psychologist and are based on the Diagnostic and Statistical Manual of Mental Disorders version 5 (DSM-V) criteria. Unfortunately, a number of factors complicate the nature of these diagnostic assessments. These include the poor general understanding of the neurobiological underpinnings of psychiatric disorders, such as PTSD and major depressive disorder (Pinna, 2018). These conditions are multifaceted and heterogenic for symptoms and for the way they manifest in different patients. The finding that symptoms overlap and comorbidity among various psychiatric disorders, including depression, anxiety, substance abuse and suicide, further complicates diagnosis (Locci and Pinna, 2017; Franklin et al., 2018). Objective neurobiological parameters are not yet in the clinical practice unlike in the diagnosis of most of the medical conditions. In recent years, several biomarker candidates have been suggested for PTSD, however, their diagnostic value remains to be yet established (reviewed in Aspesi and Pinna, 2018). As in the symptoms and comorbidity of mood disorders, these biomarkers for PTSD are often common to other neuropsychopathologies, such as major depressive disorder. For example, downregulation of neurosteroid biosynthesis, including the concentrations of the GABAergic endogenous modulator allopregnanolone and of its equipotent stereoisomer, pregnanolone was found in cerebrospinal fluid (CSF), plasma, serum of major depression and PTSD patients (Romeo et al., 1998; Uzunova et al., 1998; Rasmusson et al., 2006, 2019; Pineles et al., 2018). In PTSD patients, CSF allopregnanolone levels inversely correlated with levels of dehydroepiandrosterone (DHEA), likely generating an imbalance between inhibitory and excitatory neurotransmission underlying PTSD symptoms (Rasmusson et al., 2006). Importantly, sleep disturbance in the context of PTSD was previously associated with DHEA responses following adrenal activation as well as with decreased allopregnanolone levels (reviewed in Pitman et al., 2012). The significance of allopregnanolone biosynthesis downregulation as a biomarker of psychiatric disorders has been highlighted in numerous reports (Uzunova et al., 1998; Nemeroff, 2008; Agis-Balboa et al., 2014; Dichtel et al., 2018; reviewed in Schüle et al., 2011; Zorumski and Mennerick, 2013; Schüle et al., 2014; and Locci and Pinna, 2017). Neurosteroid biosynthesis deficit observed in PTSD patients has been successfully modeled in rodents subjected to chronic stress, such as in mice exposed to prolonged (3–4 weeks) social isolation stress.

Clinical and preclinical observations suggest that allopregnanolone may serve as a *biomarker* for symptoms overlapping in neuropsychopathologies encompassing from PTSD and depression (Pibiri et al., 2008; Pinna et al., 2008; Pinna and Rasmusson, 2012; Locci and Pinna, 2019b). In this respect, the synergic interplay of multiple neurochemical alterations that have been newly proposed within neurosteroid levels, their receptors and biosynthetic enzymes, as possible biomarkers, which is, establishing a *biomarker axis* may be the most accurate path to predict, diagnose, prevent or treat mood disorders (discussed in Aspesi and Pinna, 2018).

These summaries also suggest that by counteracting the downregulation of allopregnanolone biosynthesis, novel treatment may ameliorate symptoms in PTSD and depression (Rupprecht, 2003; Rupprecht et al., 2009, 2010; reviewed in Locci and Pinna, 2017). Indeed, allopregnanolone biosynthesis promises to be instrumental for a much-needed precision medicine for mood disorders (Aspesi and Pinna, 2018).

## Animal Models of PTSD

Establishing reliable biomarkers and specific treatments for PTSD has been hampered not only by the relative difficulty in establishing PTSD animal models but also because of the limited knowledge on PTSD neurobiology (Borghans and Homberg, 2015; Pinna and Izumi, 2018; Aspesi and Pinna, 2019). However, establishing correlative analyses among altered neuroactive chemicals in patients’ plasma, serum, and CSF is key to translate findings to animal models. Animal models are essential investigative tools to understand the etiopathology of a disease/disorder, how this develops over time and what targets can be affected by new pharmacological treatments. While it is beyond impossible to precisely model complex behavioral expressions of human symptoms that recapitulate to PTSD, basic behavioral endophenotypes can be reproduced in animals (reviewed in Siegmund and Wotjak, 2006, 2007). At this regard, animal models must satisfy criteria including *face, construct* and *predictive validity* Geyer and Markou, 2002). *Face validity* is the collection of phenotypes (behavioral and neurochemical) that relate finding in PTSD patients to rodent stress or genetic models. *Construct validity* is the process involved in the onset and the manifestation of the disorder and this, ultimately, is recapitulated in the animal model. Finally, p*redictive validity* reflects the capability of animals to inform by means of predictors on the human disorder.

Probably the most commonly used stressful experimental condition to elicit stress-induced behavioral deficits that recapitulate to PTSD symptoms includes *the restraint stress*. Rodents are generally restraint under one single exposure that may last up to 2 h (Whitaker et al., 2014) or during repeated sessions that vary from few days to several weeks (Gameiro et al., 2006).

Pairing the restraint stress with forced swimming and other stressors is part of *the unpredictable variable stress*, which reproduces PTSD behavioral deficits that are ameliorated by administration with SSRIs or ketamine (Garcia et al., 2009; Yin et al., 2016). This procedure is believed to model the unpredictable stress that soldiers often experience in warzones (Wakizono et al., 2007; Goswami et al., 2013; Shepard et al., 2016). In addition to a PTSD-like phenotype, the unpredictable protocol is associated with depressive-like deficits typically observed in PTSD patients with comorbidity with depression.

*The inescapable shocks* is another unpredictable stressor-based model, which relies on an unexpected single stress-exposure, an electric foot or tail shock and is generally used to model fear responses and fear extinction learning (Pryce et al., 2011; Desmedt et al., 2015). The inescapable shock model can be combined with restraint (Nagata et al., 2009).

*The predator-stress model* protocol includes the exposure of rodents to a predator or to its scent (Adamec et al., 2004; Wilson et al., 2014a). This stressor induces hyperarousal, avoidance, fear, and reduces fear extinction (Cohen et al., 2010; Zoladz et al., 2015; Seetharaman et al., 2016). Exposure to predators also increases anxiety-like behavior (Adamec et al., 2005). Behavioral deficits are heightened when rodents are directly exposed to a predator rather than the predator scent. These animals also respond to sertraline, which reduces anxiety-like behavior and cue avoidance (Zoladz et al., 2013; Wilson et al., 2014b).

*The single prolonged stress* consists in three stressors that are administered in succession: restraint stress (2 h), forced swimming (20 min) and exposure to diethyl ether (Liberzon et al., 1997, 1999). Cue-conditioned fear and its extinction are unaffected; however, this procedure induces consistent impairment in extinction retention (George et al., 2015). This model also induces hyperarousal and enhanced contextual freezing (Imanaka et al., 2006; Yamamoto et al., 2009). Cue-induced fear can be attenuated by paroxetine (Perrine et al., 2016).

*The social defeat stress* model is mostly performed in male rodents by a resident-intruder test, which results in aggressive behavior and social stress for the intruder (Björkqvist, 2001; Hammels et al., 2015). This increases social avoidance and other behavioral traits of PTSD, including hyperarousal and anhedonia (Warren et al., 2013; Der-Avakian et al., 2014).

The 129S1/SvlmJ genetic mouse model of PTSD (Camp et al., 2009) is characterized by impaired fear extinction (Hefner et al., 2008). Importantly this model allows investigating the molecular and genetic mechanisms underlying fear extinction from a genetic perspective allowing studies on individual vulnerability, as well as, their predisposition to PTSD. Similarly to most of PTSD rodent models reviewed above, the 129S1/SvlmJ mouse also responds to SSRIs, such as fluoxetine that improves the fear responses (Camp et al., 2012).

Finally, serotonin 2C receptors (5-HT2CR) are well characterized in anxiety, and a new model in mice having the fully VGV edited isoform of 5-HT2CR, which overexpresses brain 5-HT2CR, was recently established to study PTSD predisposition (Règue et al., 2019). VGV mice expressed greater fear responses, fear extinction deficits, and fear generalization. These dysfunctions were normalized by paroxetine in VGV mice given acutely and decreased when administered chronically. This treatment also improved deficits in brain derived neurotropic factor (BDNF) expression in the amygdala and the hippocampus. VGV-transgenic mice express neurobiological features relevant to PTSD and its treatment (Règue et al., 2019).

By far, “PTSD model” has often been an overused terminology to depict basic research studies that include a number of stressors to induced behavioral deficits (Siegmund and Wotjak, 2006). The human condition should probably be modelled by applying an uninterrupted chronic stress in combination with an acute traumatic event. Generally, the first serves an essential substrate for “trauma/fear incubation” and the second is a trigger that challenges the individual susceptibility to develop resilience or PTSD symptoms. However, reproducing chronic stress in animal models is a hard task in that most paradigms administer repeated acute stressors, which results in an intermittent stress model. Protracted social isolation stress may offer an alternative to this methodological problem and provide the advantage of administering the chronic stressor continuously and for as long as desired (often weeks; reviewed in Zelikowsky et al., 2018). This phase of neurochemical changes, such as social isolation stress-induced neurosteroid biosynthesis downregulation, may provide the required conditions that precipitate PTSD-like behavior following the administration of acute stressors (i.e., foot shocks that are part of the fear conditioning paradigm; Torok et al., 2018).

## The Socially Isolated Mouse

The protracted social isolation stress, in humans, called perceived social isolation (PSI) or loneliness, elicits a number of physical, neurological and psychological deficits that range from Alzheimer’s disease to major depression, anxiety disorders and suicidality (Cacioppo and Cacioppo, 2016). Social and community support is fundamental for emotional regulation following traumatic stress, their absence puts at risk for PTSD and other mental disorders (Nemeroff et al., 2006; Charuvastra and Cloitre, 2008; Mehnert et al., 2010). An individual inability to manage emotional memories often results in avoidance, re-experiencing symptoms and hypervigilance (Cahill et al., 2003; Rothbaum and Davis, 2003; Pitman et al., 2006; Rauch et al., 2006).

Rodents that have been exposed to a prolonged *social isolation* in individual cages for 3–4 weeks, express time-dependent behavioral deficits, including increased anxiety-like behavior and aggression (Guidotti et al., 2001; Pinna et al., 2003; Rau et al., 2005; Pibiri et al., 2008; discussed in Locci and Pinna, 2019b). Individual housing is likewise a powerful stressful condition that may increase the susceptibility to develop behavioral dysfunctions when rodents are additionally exposed to an acute traumatic stressor, for example, the electric shocks that constitute the fundamental of the Pavlonian fear conditioning test (Charuvastra and Cloitre, 2008; Pinna, 2010).

Behavioral deficits following protracted social isolation are associated with a number of physical and neuronal dysfunctions, including impairment of the HPA axis, neurotransmitter systems, neuropeptides, neurohormones, and neurotropic factors (reviewed in Nin et al., 2011a). Importantly, studies have investigated the potential role for tachykinins in regulating social isolation-induced aggression in mice. Studies focusing on the neuropeptide tachykinin 2 (Tac2)/neurokinin B (NkB) showed that in the central amygdala the peptide plays a role in fear memory consolidation. A more recent study showed that Tac2/NkB is dramatically upregulated throughout the brain following protracted social isolation, which resulted in aggression and impairment of other behaviors by acting on multiple brain regions (Zelikowsky et al., 2018).

Studies from this lab have mainly focused on the effects of social isolation on the GABAergic neurotransmission dysfunction caused by impaired neurosteroid biosynthesis, and changes in the expression of several GABA_A_ receptor subunit subtypes. The role of neurosteroids in regulating the expression of neurotropic factors (i.e., BDNF) during social isolation has also been one important research interest.

### Behavioral Deficits in Socially Isolated Mice

**Figure 2:**
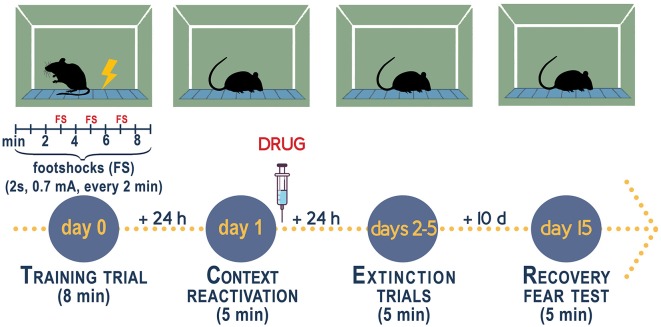
Experimental procedure to measure fear conditioning responses, fear extinction, and fear extinction retention in socially isolated mice. Contextual fear conditioning responses in socially isolated is studied after 4 weeks of isolation when the decline of allopregnanolone is maximal (Pibiri et al., 2008; Pinna et al., 2008). Group-housed mice of the same age as the socially isolated mice serve as control. Socially isolated mice express a decrease of corticolimbic allopregnanolone levels that is associated with an enhancement of contextual fear responses and impaired fear extinction (Pibiri et al., 2008). The fear-conditioning apparatus, which schematized in the figure, consists of a transparent acrylic chamber measuring 25 cm wide, 18 cm high, and 21 cm deep (San Diego Instruments). The cage floor is composed of stainless-steel rods connected to an electric shock generator. A small fan is located on the top wall of the enclosure. The chamber is surrounded by a frame with 16 infrared photo beams. A computer controls the delivery of electric foot shocks and auditory stimuli and records beam interruptions and latencies to beam interruptions (freezing time). *Training Test*. During the training, mice are placed into the training chamber and allowed to explore it for 2 min. After this time, they receive an unconditioned stimulus (US, electric footshock, 2 s, 0.5 mA). The footshock is repeated three times every 2 min. After the last tone plus shock delivery, mice are allowed to explore the context for an additional minute before removal from the training chamber (total of 8 min). *Contextual Test*. Twenty-four hours after training, the mice are placed in the contextual cage, and freezing behavior is measured for 5 min (Freeze Monitor System, San Diego Instruments) without footshock presentation. *Extinction Test*. For contextual extinction experiments, mice are placed in the contextual cage for 5 consecutive days starting 24 h after the training session. *Fear extinction retention*. Retention of fear extinction is measured by placing the mice to the context for 5 min following an interval of 10 days. Freezing behavior is measured for 5 min without tone or footshock presentation. Freezing is defined by the absence of any movement except for those related to respiration while the animal is in a stereotypical crouching posture (Pibiri et al., 2008). To disrupt aversive memories through a reconsolidation blockade (Stern et al., 2012), drugs are given immediately after a contextual fear conditioning reactivation session (Pinna and Rasmusson, 2014; Locci and Pinna, 2019b).

Mice that are socially isolated for 3–4 weeks post-weaning (PN21) express a number of behavioral deficits relevant to model aspects of human mood disorders (reviewed in Pinna and Rasmusson, 2012; Zelikowsky et al., 2018; Aspesi and Pinna, 2019; Locci and Pinna, 2019b). Specifically, male mice when exposed to a fear conditioning test with administration of a conditioned (CS, acoustic tone) and an unconditioned stimuli (US, footshock, please see Figure 2), in a novel context, comprising a contextual chamber (Pibiri et al., 2008; Pinna et al., 2008), show elevated freezing, which is an index of elevated fear responses, 1 day post-training session. Time-course experiments have unveiled that freezing increases time-dependently during 4 weeks of isolation and similarly to the expression of aggressive behavior, reaches a plateau between week 4 and 6 of isolation (Pibiri et al., 2008; Pinna et al., 2008). In this mouse model of enhanced fear responses, socially isolated mice exhibit an impaired fear extinction memory as compared with group-housed control male mice and a re-emergence of fear after the passage of time or, in other words, they show impaired fear extinction retention (Pibiri et al., 2008). On a translational standpoint, social isolation can be seen as a prolonged stress that is often associated with a precipitating traumatic event, which leads to maladaptive post-stress adaptations and emergence of PTSD in patients. Thus, social isolation offers a suitable model to study vulnerability to PTSD (discussed in Aspesi and Pinna, 2019).

Other behavioral deficits expressed by socially isolated mice include increased aggression to a same-sex intruder, as well as, anxiety-like and depressive-like phenotypes. These behavioral traits are consistent with behavioral aspects that are reminiscent of PTSD symptoms often observed in PTSD patients following re-exposure to trauma reminders (Grillon and Morgan, 1999; Rauch et al., 2006). Limitations of this animal model include studies that were conducted mostly in male mice. Socially isolated female mice investigation was mostly limited to the study of depressive-like behavior (Weiss et al., 2004; Grippo et al., 2007).

A number of pharmacological agents, including SSRIs administered at low doses that act like *selective brain steroidogenic stimulants* (SBSSs) and increase corticolimbic allopregnanolone levels (Pinna, 2015), or allopregnanolone analogs, including ganaxolone, by a contextual fear reconsolidation blockade, normalize fear response and facilitate fear extinction (Pibiri et al., 2008; Pinna and Rasmusson, 2014; Rasmusson et al., 2017). Most importantly, these agents prevent the reemergence of fear after the passage of time, during recall (Pinna and Rasmusson, 2014; reviewed in Aspesi and Pinna, 2019; Locci and Pinna, 2019a; Raber et al., 2019). Furthermore, the novel allopregnanolone’s analogs BR297 and BR351 showed strong anti-aggressive effects in isolated mice (Locci et al., 2017). Another strategy to increase allopregnanolone levels and enhance activation of emotion regulation neurocircuits includes administration with the allopregnanolone precursor pregnenolone (Sripada et al., 2013). Recently, neurosteroidogenic agents, including the endocannabinoid-like, PEA by a similar mechanism, which include upregulation of allopregnanolone biosynthesis, showed to improve fear extinction and its retention in socially isolated mice compared to non-stressed mice (Locci and Pinna, 2019b). PEA also decreased anxiety-like and depressive-like behavior and aggression in socially isolated mice (Locci et al., 2017; Locci and Pinna, 2019b). Recently, by directly manipulating the endocannabinoid system by administering the endocannabinoid reuptake inhibitor AM404 facilitated safety learning in a CB1-dependent manner and attenuated the relapse of avoidance (Micale et al., 2017). Although a direct evidence that endocannabinoids stimulate brain neurosteroid biosynthesis has not been provided, recent studies show THC increases allopregnanolone’s precursor, pregnenolone by activating CB1 (Vallée et al., 2014; Vallée, 2016). The detailed description on endocannabinoid and neurosteroidogenic neuronal targets and novel molecules that are currently investigated for the development of new treatments for PTSD has been the focus of recent reviews (Pinna, 2014; Aspesi and Pinna, 2019; Locci and Pinna, 2019a; Raber et al., 2019).

### GABA_A_ Receptor Subunit Expression and Benzodiazepine Inefficacy in Socially Isolated Mice

Altered corticolimbic GABAergic neurotransmission, including GABA_A_ receptor subunit composition have been linked with a number of mental disorders (Akbarian et al., 1995; Dean et al., 1999; Lewis, 2000; Ishikawa et al., 2004). Affinity for the benzodiazepine binding at GABA_A_ receptors is strongly dependent on α1–3,5 and γ2 subunits (Rudolph et al., 1999; Rudolph and Möhler, 2004). Intriguingly, GABA_A_ receptor subunit expression is highly susceptible to stress effects, pharmacological interventions, as well as, alcohol and substance abuse (Impagnatiello et al., 1996; Pinna et al., 2006a; Bohnsack et al., 2017, 2018; Locci and Pinna, 2017). Protracted stress induces profound changes in the expression of GABA_A_ receptors that alters the receptor sensitivity to endogenous modulators and synthetic agonists (reviewed in Locci and Pinna, 2017). In socially isolated mice, the mRNA and protein expression of α1, α2, and γ2 of the GABA_A_ receptor subunits were found reduced by 50% when compared to those of control group-housed mice (Pinna et al., 2006a; Nin et al., 2011b). The expression of α4 and α5 subunits was instead over-expressed by 130% (Pinna et al., 2006a). Protein expression of α1 and α5 in frontal cortices and hippocampal synaptic membranes were likewise decreased and elevated, respectively (Pinna et al., 2006a; reviewed in Locci and Pinna, 2017). Studies at the cortical layer- and cell-specific levels showed that in laser microdissected frontocortical layer I, expression of α1 subunit was decreased by 50% and it was unchanged in the layer V pyramidal neurons following social isolation (Pinna et al., 2006a).

Behavioral pharmacological studies showed that socially isolated mice exhibit a robust resistance to the sedative and anxiolytic pharmacological properties of diazepam and zolpidem. These synthetic agonists act at GABA_A_ receptor-containing α1–3, 5 subunits (Pinna et al., 2006a). Thus, α1 and 2 subunit downregulation *per se* may explain the decreased responsiveness of socially isolated mice to sedative and anxiolytic benzodiazepines. These results further suggest that γ2 subunit downregulation may have originated a switch with γ subunits that are largely expressed in extrasynaptic GABA_A_ receptors with a loss of benzodiazepine binding sites that was determined in cortical synaptosomes (Pinna et al., 2006a). Hence, prolonged stress may be associated with formation of benzodiazepine-insensitive GABA_A_ receptors in cortical neurons that modulate anxiolytic responses (Rudolph et al., 1999; Rudolph and Möhler, 2004; Nin et al., 2011b).

Intriguingly, increases in α4 and δ-subunits in frontocortical membranes from socially isolated rodents (Pinna et al., 2006b; Serra et al., 2008) may originate GABA_A_ receptors for which endogenous modulators, including allopregnanolone, show a stronger affinity (Belelli and Lambert, 2005; Belelli et al., 2005). Actually, allopregnanolone administered to socially isolated mice induces anxiolytic effects (Pinna et al., 2008).

Translationally, GABA_A_ receptor expression in the socially isolated mouse shows several commonalities with PTSD patients. Indeed, stress-induced remodeling of GABA_A_ receptors in PTSD patients results in loss of benzodiazepine pharmacological actions due to decreased benzodiazepine-binding sites to cortex, hippocampus, and thalamus (Geuze et al., 2008). These preclinical and clinical findings provide support for the observation that treatment with benzodiazepine is ineffective for PTSD treatment and prevention. Furthermore, risks associated with their administration generally outweighs the short-term benefits. Benzodiazepine use in the general population is associated with adverse effects (tolerance, dependence and withdrawal symptoms), in patients with PTSD side effects are even more severe and a study showed significantly increased risk of developing PTSD with their use after recent trauma, worse psychotherapy outcomes, aggressiveness, depression symptoms, and substance use (Deka et al., 2018). In another study, veterans with PTSD administered with benzodiazepines showed higher rates of health care utilization and were more likely to attempt and complete suicide (Guina et al., 2015). Benzodiazepines are, thus, contraindicated for patients with PTSD or recent trauma, evidence-based treatments for PTSD should be favored.

### Allopregnanolone Downregulation and Fear Circuitry in Socially Isolated Mice

Allopregnanolone biosynthesis has been found altered in several mood disorders, including depression, anxiety, PTSD, post-partum depression and premenstrual syndrome (Romeo et al., 1998; Uzunova et al., 1998; Rasmusson et al., 2006, 2019; Nemeroff, 2008; Lovick, 2013; Dichtel et al., 2018; Pineles et al., 2018). This deficit was more recently observed in the fronto-cortical pyramidal neurons of the Broadman area 9 (BA9) of male patients affected by major depression (Agis-Balboa et al., 2014). As previously mentioned, therapeutically, elevating the down-regulated allopregnanolone levels in patients with mood disorders also correlated with improved patients’ symptoms (Romeo et al., 1998; Uzunova et al., 1998; Agis-Balboa et al., 2014; Kanes S. J. et al., 2017; Kanes S. et al., 2017).

In socially isolated rodents the responsiveness of the HPA axis is decreased. Levels of corticosterone and release of CRH are decreased in the blood flow (Sanchez et al., 1998; Chida et al., 2005; Malkesman et al., 2006). The HPA axis hypo-function is even more evident when socially isolated rodents are exposed to acute stressors. This finding underlies an overall reduced sensitization of the HPA axis to acute stressful stimuli (Sanchez et al., 1998). In rodents, corticolimbic neurons express the biosynthetic enzymes, 5α-reductase type I and 3α-HSD that synthesize allopregnanolone (Agís-Balboa et al., 2006, 2007). Consistently, socially isolated rodents show a time-dependent impairment of neurosteroidogenesis, including the levels of the GABAergic neurosteroid, allopregnanolone. This deficit has been associated with appearance of a number of behavioral dysfunctions, such as delayed and incomplete fear extinction and reemergence of fear upon fear recall (Pibiri et al., 2008; Pinna and Rasmusson, 2014) that resemble behavioral deficits showed in patients affected by anxiety, depressive disorders, and PTSD (Matsumoto et al., 1999; Pinna, 2010; Schüle et al., 2011).

For over a decade, investigating the neurochemical and behavioral deficits expressed by socially isolated rodents, this laboratory, as well as other colleagues, have established that either rats or mice that undergo individual caging, which results in a form of prolonged stress for several weeks, express a downregulation of allopregnanolone levels in corticolimbic areas. This is maintained and results from a downregulation of the expression of 5α-reductase type I, a rate-limiting enzyme in allopregnanolone biosynthesis (Matsumoto et al., 1999; Serra et al., 2000; Pinna et al., 2003; Bortolato et al., 2011; reviewed in Matsumoto et al., 2007). The biosynthesis rate of allopregnanolone and its precursor, 5α-DHP in socially isolated is decreased by 70% when compared to that of group-housed mice (Dong et al., 2001; Pinna et al., 2003). New finding also shows that socially isolated mice express a downregulation of P450scc, another rate-limiting enzyme involved in the inner mitochondrial membrane metabolism of pregnenolone from cholesterol (Locci and Pinna, 2019b). Across several brain areas analyzed, 5α-reductase largest expression decrease was observed in the amygdala and hippocampus (Agís-Balboa et al., 2007). The olfactory bulb and the frontal cortex expressed a moderate downregulation in the neurosteroid biosynthetic enzymes. Importantly, 5α-reductase type I expression did not change in the cerebellum and striatum (Agís-Balboa et al., 2007). As revealed by *in situ* immunohistochemical experiments, 5α-reductase was specifically downregulated in layers V–VI cortical pyramidal neurons, in hippocampal CA3 pyramidal neurons and in dentate gyrus glutamatergic granular cells as well as pyramidal-like neurons of the basolateral amygdala (Agís-Balboa et al., 2007). Importantly, 5α-reductase expression was not decreased in GABAergic long-projecting neurons of the reticular thalamic nucleus, central amygdala, cerebellum, and in the striatum medium spiny neurons. This enzymatic expression decrease was paralleled by a decreased allopregnanolone in discrete corticolimbic areas that was quantified by GC-MS, characterized by unsurpassed structural selectivity and sensitivity (Pibiri et al., 2008; Locci and Pinna, 2019b).

**Figure 3:**
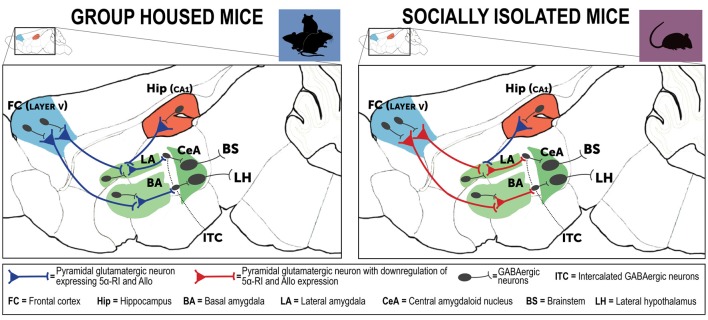
Neurocircuitry underlying the PTSD-like phenotype expressed by socially isolated mice. This is a simplified schematic representation of mouse brain neurocircuitry regulating emotional behavior under physiological (group-housed) and stress-induced deficits (social isolation). The prefrontal cortex and hippocampus directly project to the amygdaloid nuclei to regulate their hyperactivity following traumatic events (Herry et al., 2008). In susceptible individuals, a stressful experience is associated with impairment of cortical inhibitory activity directed to the amygdala, which results in exaggerated hyperactivity and inappropriate fear responses (Akirav and Maroun, 2007; Raber et al., 2019). In PTSD, amygdala hyperactivity is part of a maladaptive emotional processing resulting from exposure to traumatic events. The neural substrates of these behavioral deficits may result from decreased GABA release (downregulated allopregnanolone concentrations (Rasmusson et al., 2006, 2019), in participation with changes in GABA_A_ receptor subunit subtypes (Geuze et al., 2008). Collectively, these neurobiological alterations may explain emergence of PTSD symptoms (Pinna, 2018). In the socially isolated mice, a stress-induced model of PTSD-like behavioral traits, cortical and hippocampal projections directed to the basolateral amygdala (BLA) show a downregulation of allopregnanolone biosynthesis and behavioral correlates, including increased fear responses and impairment of fear extinction (Agís-Balboa et al., 2007; Pinna et al., 2009). In socially isolated mice (*right panel*), allopregnanolone downregulation in cortical and hippocampus pyramidal glutamatergic neurons and in pyramidal-like neurons of the BLA may represent the molecular underpinnings that recapitulate an increased excitability of the neuronal pathway that converges to the intercalated GABAergic neurons (ITC) and central amygdala (CeA) GABAergic spiny neurons (Agís-Balboa et al., 2007; Pinna et al., 2008). Collectively, reduction of allopregnanolone biosynthesis in corticolimbic glutamatergic neurons may impair cortico-hippocampal-amygdaloid circuits by inhibiting the GABAergic output neurons of the CeA, which project to the hypothalamus and brainstem and may explain the excessive fear responses and other behavioral deficits observed in socially isolated mice (Pinna et al., 2008, 2009). Allo, allopregnanolone; 5α-RI, 5α-reductase type I.

These findings underlie and sustain a dysfunction in corticolimbic circuits that in socially isolated mice is responsible for behavioral deficits (Figure 3). Indeed, amygdala pyramidal-like neurons are involved in the regulation of the strength of the inhibitory function of the intercalated inhibitory spiny GABAergic interneurons (ITC) that mediate the connectivity between the basolateral amygdala (BLA) and the central amygdaloid nucleus (CeA; Agís-Balboa et al., 2007). One of the most replicated traits of PTSD connectivity studies is the typical exaggerated amygdala hyperactivity, which results from functional deficits of projections from the prefrontal cortex and hippocampus (Akirav and Maroun, 2007). These glutamatergic neurons located in the prefrontal cortex and hippocampus extend and synapse on GABAergic neurons of the amygdala and regulate an inhibitory input to these amygdala neurons (depicted in Figure 3). In normal individual or in resilient subjects, fear following traumatic events can be suppressed by the regulatory role exerted by the prefrontal cortex and hippocampus projections that directly synapse with the amygdala and shut down its hyperactivity. In maladaptive conditions following a traumatic event, this process can be impaired and the cortical inhibitory function on the amygdalar nuclei may be weakened, which results in amygdala hyperactivity and inappropriate and exaggerated fear response and impaired fear extinction, a core neurobiological trait observed in PTSD (Liberzon and Sripada, 2008). Hence, prefrontal cortex regulation of the amygdala ITC neurons dictates the responsiveness to stress and fear (Pare et al., 2004). These GABAergic outputs exert a pivotal role in emotion regulation following stress and directly influence fear extinction learning and regulate the CeA output that mediates responses to conditioned fear (Likhtik et al., 2008). Several lines of evidence have shown that ITC neuron lesions impair fear extinction memory, while activation of these neurons facilitates extinction learning (Jüngling et al., 2008; Likhtik et al., 2008). ITC GABAergic and CeA projections to brainstem and hypothalamus modulate fear responses and fear extinction following stressful events (Pinna et al., 2009). Altogether, the corticolimbic circuits that in socially isolated mice express downregulated allopregnanolone levels, which include the prefrontal cortex, hippocampus and amygdala are directly responsible for the expression of emotional behaviors, including aggressive behavior, fear responses, and anxious behavior, which are commonly observed in PTSD patients (LeDoux, 2000; Milad et al., 2007). In socially isolated mice, these deficits in allopregnanolone biosynthesis and the behavioral dysfunction have been associated with a decrease of corticolimbic BDNF expression (Nin et al., 2011a).

It is important to note that most of the studies in humans with major depressive disorder and PTSD have determined levels of allopregnanolone in the periphery (serum, plasma, CSF) and only a few have quantified levels of allopregnanolone in the post-mortem brain (Agis-Balboa et al., 2014; Cruz et al., 2019). Oppositely, animal studies have for the most part focused on allopregnanolone levels in specific brain regions (Pibiri et al., 2008; Pinna et al., 2008; Locci and Pinna, 2019b). Brain levels of allopregnanolone may also influence the HPA and HPG axes. For instance, the HPA axis can be modulated by the neuronal inhibition initiated by GABAergic neurons within the hypothalamus. Corticosteroids exert a negative feedback on the HPA axis by acting on the hippocampus and the medial prefrontal cortex, which triggers a spike-dependent elevation in GABA release from inhibitory synapses thus stimulating the function of GABAergic neurotransmission. By this mechanism, allopregnanolone may also induce a potent inhibition on the HPA axis activity, which attenuates plasma ACTH and corticosterone increase induced by stress. Thus, locally brain produced allopregnanolone may contribute to regulating neuronal function by modulating HPA axis activity (reviewed in Biggio et al., 2014).

## Transdiagnostic Behavioral Features of the Socially Isolated Mouse

### PTSD/Suicide

The decrease of allopregnanolone in socially isolated mice has been associated with behavioral deficits, including anxiety-like behavior and aggression. Further, socially isolated mice show impairment of fear extinction and spontaneous reemergence of fear following passage of time and determined during a recall session (Pibiri et al., 2008; Pinna and Rasmusson, 2014; Locci and Pinna, 2019b). Probably, one of the most remarkable behavioral deficits of socially isolated mice regards the heightened aggressive behavior of a resident socially isolated mouse towards a same-sex intruder (Pinna et al., 2003). It is intriguing to note that the expression of aggressive behavior is one prominent behavioral phenotype used to model behavioral traits of suicide occurring in men. If one considers that PTSD is often complicated by comorbid suicidal ideation and suicide attempts, the socially isolated mouse may entail important transdiagnostic features to model aspects seen in the spectrum of PTSD-associated with suicide risk, often observed in veterans (this aspect was recently reviewed in Locci and Pinna, 2019b).

### PTSD/AUD

AUD has a general high prevalence in the American population and has even higher abuse rates within PTSD patients (Blanco et al., 2013; Debell et al., 2014; Shorter et al., 2015). Alcohol consumption in subjects with psychiatric conditions is often practiced as a form of self-medication. While substance use disorder is reported to be about double among PTSD patients, AUD reached a 4-fold higher prevalence than the general population, which makes alcohol the most abused substance between PTSD individuals (Jacobsen et al., 2001). Studies in children victims of sexual, psychological and physical abuse have evidenced the higher lifetime prevalence of AUD and PTSD symptoms (Khoury et al., 2010). Comorbidity of PTSD with AUD is even more increased among military personnel (Gates et al., 2012). Progress in understanding the neurobiology of this severe and impactful comorbidity has generally been impeded by the paucity of animal models of PTSD/AUD.

Social isolation in rodents has been often used as a model to predict risk factor for both PTSD and AUD (recently reviewed in Gilpin and Weiner, 2017). Indeed, the social isolation protocol steadily increases ethanol self-administration in a number of methodological procedures, including consumption of ethanol vs. sucrose in a limited-access intermittent two-bottle choice paradigm. Alcohol intake and preference were reported to increase up to 8 weeks (Skelly et al., 2015). Another research team that has used social isolation in male Sprague-Dawley rats during PD 21–42 observed the same results. In this model, conditioned place preference for alcohol was increased (Whitaker et al., 2013), but this model failed to lead to long-lasting anxiety-like behavior or elevated alcohol drinking in females Long Evan rats (Butler et al., 2014). While most experiments were conducted using rats, similar behavioral patterns were noted when mice were isolated in adolescence, which is also associated with more prominent emotional behavioral deficits, such as aggression, sensory gating and fear deficits (Pibiri et al., 2008; Koike et al., 2009; Gan et al., 2014; Kumari et al., 2016; Locci et al., 2017). Home-cage elevated alcohol consumption and preference that lasted even 1 month during adulthood was primarily observed in male socially isolated mice (Advani et al., 2007; Lopez et al., 2011; Talani et al., 2014).

Several lines of evidence suggest vulnerability to comorbid PTSD and AUD results from sensitization of the dopaminergic mesolimbic system and specifically, decreased dopamine in nucleus accumbens and elevated responsivity of the dopaminergic circuitry connecting VTA–NAc may underlie comorbidity of PTSD and AUD (reviewed in Gilpin and Weiner, 2017). However, other findings have shown that hippocampus allopregnanolone levels are associated with downregulation in hippocampal synaptic excitability and LTP in socially isolated rats (Serra et al., 2000; Pibiri et al., 2008; Sanna et al., 2011; Talani et al., 2016; Locci and Pinna, 2017).

Collectively, social isolation during adolescence appears as a critical period to increase susceptibility to both traumatic stress-induced alcohol drinking and emotional deficits relevant with symptoms of PTSD in humans (Pibiri et al., 2008; Pinna et al., 2008; McCool and Chappell, 2009; Skelly et al., 2015; Locci and Pinna, 2017). Furthermore, these behavioral effects are heightened in male rodents (Butler et al., 2014).

## Conclusion

**Figure 4:**
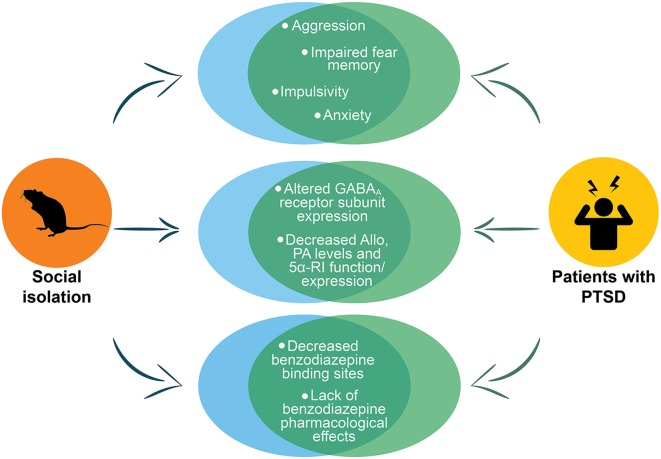
Representation of the main PTSD phenotypes in socially isolated mice. The figure shows the phenotypes in socially isolated mice that recapitulate to deficits in PTSD patients. The circles at the top of the figure show the behavioral phenotype of socially isolated mice that includes aggression, impaired fear memory, impulsivity and anxiety, which are all core behavioral symptoms of PTSD. In the center of the figure are reported the abnormalities in GABA_A_ receptors and neurosteroid levels. The last circles at the bottom report the altered pharmacological response to benzodiazepines that has been reported both in socially isolated mice and in humans with PTSD. Allo, allopregnanolone; PA, pregnanolone; 5α-RI, 5α-reductase type I.

PTSD rodent models are far from optimal because they only partially reproduce phenotypic expression of PTSD neurobiology. The symptoms overlap and comorbidity among several mental disorders (e.g., PTSD, depression, anxiety, AUD and suicide) make even more challenging assessing a PTSD preclinical model. The socially isolated mouse model recapitulates several aspects of PTSD neurobiology, including downregulated corticolimbic allopregnanolone concentrations, changes in GABA_A_ receptor subunit composition, lack of benzodiazepine pharmacological action, and altered neurocircuitry of fear (summarized in Figure 4). These neurochemical alterations are associated with a number of behavioral dysfunctions that are core traits of PTSD, including heightened fear responses and impaired fear extinction, as well as, transdiagnostic behavioral features such as, elevated aggressiveness, a behavioral trait that predicts suicide, depressive- and anxiety-like behavior and increased alcohol consumption. Thus, the socially isolated mouse may reproduce a two-hit PTSD/AUD model as well as a PTSD/suicide model (Locci and Pinna, 2019a), comorbidities, which are consistently observed in PTSD patients. These features make the socially isolated mouse a suitable model to study new pharmacological approached as well as establishing a *biomarker axis* for PTSD and PTSD with comorbid AUD or suicide.

## Author Contributions

The author confirms being the sole contributor of this work and has approved it for publication.

## Conflict of Interest Statement

The author declares that the research was conducted in the absence of any commercial or financial relationships that could be construed as a potential conflict of interest.

## Footnotes

^1^http://investor.sagerx.com/news-releases/news-release-details/sage-announces-pivotal-phase-3-trial-status-sage-217-major
